# Causes, Effects and Methods of Monitoring Gas Exchange Disturbances during Equine General Anaesthesia

**DOI:** 10.3390/ani11072049

**Published:** 2021-07-09

**Authors:** Elżbieta Stefanik, Olga Drewnowska, Barbara Lisowska, Bernard Turek

**Affiliations:** 1 Department of Large Animals Diseases and Clinic, Institute of Veterinary Medicine, Warsaw University of Life Sciences, Nowoursynowska 100, 02-797 Warsaw, Poland; bernardturek@gmail.com; 2 National Geriatrics, Rheumatology and Rehabilitation Institute, Spartańska 1, 02-637 Warsaw, Poland; blisowska19@gmail.com

**Keywords:** ventilation, equine anaesthesia, gas exchange, hypoxemia, pulse oximetry, NIRS, respiratory monitoring

## Abstract

**Simple Summary:**

Horses are considered to be one of the most challenging domestic species to anaesthetize. Because of the compression of the abdominal visceral organs on the lungs when positioned in dorsal or lateral recumbency, general anaesthesia can cause significant changes to pulmonary function, blood circulation and gas exchange. Consequently, post-operative complications and anaesthetic mortality rates are higher for horses than for other commonly anaesthetized companion animals. There is no ideal method for monitoring respiratory gas concentrations during general anaesthesia, so it is important to know the advantages and limitations of individual methods and the factors that affect monitoring devices. The aim of this review is to summarize previously published studies regarding the causes and effects of intraoperative gas exchange disturbances as well as monitoring methods.

**Abstract:**

Horses, due to their unique anatomy and physiology, are particularly prone to intraoperative cardiopulmonary disorders. In dorsally recumbent horses, chest wall movement is restricted and the lungs are compressed by the abdominal organs, leading to the collapse of the alveoli. This results in hypoventilation, leading to hypercapnia and respiratory acidosis as well as impaired tissue oxygen supply (hypoxia). The most common mechanisms disturbing gas exchange are hypoventilation, atelectasis, ventilation–perfusion (V/Q) mismatch and shunt. Gas exchange disturbances are considered to be an important factor contributing to the high anaesthetic mortality rate and numerous post-anaesthetic side effects. Current monitoring methods, such as a pulse oximetry, capnography, arterial blood gas measurements and spirometry, may not be sufficient by themselves, and only in combination with each other can they provide extensive information about the condition of the patient. A new, promising, complementary method is near-infrared spectroscopy (NIRS). The purpose of this article is to review the negative effect of general anaesthesia on the gas exchange in horses and describe the post-operative complications resulting from it. Understanding the changes that occur during general anaesthesia and the factors that affect them, as well as improving gas monitoring techniques, can improve the post-aesthetic survival rate and minimize post-operative complications.

## 1. Introduction

Anaesthesia-related complications are responsible for the relatively high perioperative horse mortality rate, reportedly almost 1% [1], nearly 10 times higher than for dogs and cats [2]. The main reason for this is the long duration of surgery, which increases the risk of hypotension, hypoxaemia and acid–base disturbances [1,3]. Intraoperative monitoring enables the assessment of changes in physiological parameters and respiratory gases that can indicate the need for intervention to minimize the surgery-associated risks [4].

The tissue oxygen supply depends on cardiac output (CO) and the total oxygen content in arterial blood (C_a_O) [5]. Anaesthetics and the unnatural (dorsal) body position during surgery impairs respiratory gas transport [6,7], resulting in hypoventilation, ventilation to perfusion (V/Q) mismatch, diffusion limitation, shunting and reduced mixed venous oxygenation [8,9]. Many of these changes also persist into the immediate post-operative period, especially after horses are disconnected from the anaesthetic equipment and breathe on their own [9,10]. Additionally, cardiovascular disorders, such as a decrease in mean arterial blood pressure to less than 70 mm Hg and a cardiac output of 30–50 mL/kg/min, have been documented in 50% of anaesthetized horses [5].

### Chemical Regulation of Ventilation

The chemical regulation of ventilation is controlled by two chemoreceptors—central and peripheral. Stimulation of the respiratory centre in the brainstem occurs through carotid and aortic bodies, which alter their excitability as a result of a decrease in arterial oxygen partial pressure (P_a_O_2_), an increase in arterial carbon dioxide partial pressure (P_a_CO_2_) or an increase in arterial hydrogen ion (H^+^) concentration [5,11]. Chemoreceptors of the medulla oblongata are sensitive to changes in the cerebrospinal fluid pH, the level of which depends on the concentration of carbon dioxide in the blood [5,12]. Peripheral chemoreceptors are mainly responsible for the respiratory response to hypoxia, whereas hypercapnia and changes in blood pH are detected by both types of chemoreceptor [11,12].

The mechanisms regulating alveolar ventilation are no different for horses than for other species, but they are characterized by a distinctive response to changes in PaCO_2_ and PaO_2_ [5]. The physiological adaptation of the horse to exercise includes a high tolerance for arterial CO_2_, which leads to hypoxaemia due to the lack of a truly compensatory hyperventilation response during intensive physical effort [13]. There are also studies confirming that horses, compared to other animal species, have a much stronger hyperventilation response to a reduced fraction of inhaled oxygen [14]. In the study, which determined the effect of oxygen supply on PaO_2_ during recovery from general anaesthesia, a group of horses that had been insufflated showed PaO_2_ values that were significantly higher than for those that breathed atmospheric air, although the PaO_2_ in those horses rose immediately after they were moved into sternal recumbency. The study showed that in horses that breathed atmospheric air, the CO_2_ level returned to normal faster in comparison to insufflated horses, where PaCO_2_ values were significantly higher and pH remained significantly lower [11]. The authors of the study concluded that in the immediate post-operative period, horses are influenced by the depressive effects of anaesthetics on CNS chemoreceptors; consequently, the response to rising a CO_2_ level is impaired. In the recovery period, the response mechanisms may be under the influence of peripheral chemoreceptors, which are particularly sensitive to low oxygen partial pressure.

## 2. Reasons for Ventilation Impairment during Anaesthesia

The most common and important mechanisms in gas exchange disturbances are hypoventilation, atelectasis, ventilation–perfusion (V/Q) mismatch and dead space ventilation.

### 2.1. Hypoventilation

Hypoventilation is defined as an increase in partial arterial CO_2_ pressure (PaCO_2_) associated with arterial oxygen tension (PaO_2_) reduction. The primary causes of hypoventilation during surgery are central nervous system depression and respiratory muscles weakness. Hypoventilation is a result of both the reduction in minute ventilation and an increase in dead space. In anaesthetized horses, dead space can be increased by 60% due to both the position of the animal and the effect of the use of ventilation equipment [15]. In anesthetized horses, respiration is depressed, because it is influenced not only by a decreased respiratory rate, but also by a decreased tidal volume with a normal, or even increased, number of breaths [16]. The two main factors that contribute to the reduction in minute ventilation are unnatural (dorsal or lateral) recumbency [17] and the dose-dependent depressive effect of anaesthetics [18]. The diaphragm is angled more obliquely in horses than in other animals, so the lungs are mostly in a dorsal position, relative to the abdominal organs. This is not a problem in a standing horse, but in dorsal recumbency, these organs compress a large area of the lungs [17,19]. Horses also have an active phase of exhalation, which involves the abdominal muscles [20]. During anaesthesia, the active phase of exhalation is impaired; therefore, general anaesthesia combined with muscle relaxation and recumbency, especially dorsal, leads to impaired ventilation [17,19]. Hypoventilation in turn leads to an increase in CO_2_ concentration and low alveolar oxygen partial pressure (P_A_O_2_), which subsequently causes low PaO_2_ [21]. This falling P(A–a)O_2_ gradient is normal, but protracted hypoventilation may cause it to become steeper as an effect of atelectasis. Ventilation is influenced by the degree of diaphragm movement, lung compliance and chest wall movement [22]. In the decubitus position, pressure from abdominal contents leads to decreased lung compliance associated with increased airway pressure [22]. Hypoventilation caused by reduced alveolar ventilation manifested in the recovery box is one reason for the longer elimination of inhaled anaesthetics from the alveoli, and thus their prolonged effect [23].

### 2.2. Atelectasis

Atelectasis is the partial or complete collapse of a lung or an area of a lung. Atelectatic areas develop at an early stage in anesthetized horses positioned in dorsal recumbency and are the most likely cause of the large V/Q mismatch as well as the high alveolar–arterial oxygen gradient [24]. The anatomy of the horse is unique: each bronchus connects directly to the alveolar ducts; there are no respiratory bronchi, and collateral ventilation is poorly developed. Therefore, closing of a bronchus leads to the loss of an entire gas exchange unit [25]. When the area of a lung becomes occluded, a pocket of a trapped gas is created. When venous blood mixed with gas of sub-atmospheric partial pressure perfuses the pocket, where the gas partial pressure is close to atmospheric pressure, gas uptake from the pocket occurs [26]. Another mechanism takes place when the gas uptake from the alveolus into the bloodstream is higher than the gas volume entering alveolus. Development of atelectasis leads to a reduction in the functional residual capacity (FRC). Nitrogen, an insoluble gas that does not pass though the alveolar membranes, helps to create pressure inside the lungs to keep inactive alveoli open, but when supplemental oxygen is administered, it replaces the nitrogen [27]. Complete gas evacuation by diffusion leads to alveolar collapse [24]. Compression atelectasis because of organ weight is the major type of atelectasis in anaesthetized horses [6,10,19].

### 2.3. V/Q Mismatch and Shunting

In anesthetized horses in the dorsal or lateral position, the V/Q mismatch is the ultimate cause of gas exchange disturbances, leading to a large difference between alveolar and arterial oxygen concentration and eventually to hypoxemia [6,9]. It has been proven that the caudo-dorsal area of the lungs is well supplied with blood regardless of the position of the horse, while ventilation is most efficient in the uppermost parts [28]. Therefore, gas exchange is most efficient in standing horses because the area best perfused with blood coincides with the area best supplied with oxygen, whereas dorsal recumbent horses have a significant imbalance in areas of adequate ventilation and adequate blood supply. A low V/Q ratio (V/Q < 1) occurs when perfusion exceeds ventilation [21]. A low V/Q ratio leads to hypoxemia due to a decreased alveolar oxygen level, which subsequently causes a decreased arterial oxygen level. The impact on the removal of CO_2_ is minimal. Hypoxemia caused by a low V/Q ratio is easy to reverse with increased FiO_2_. A high V/Q ratio (V/Q > 1) develops when ventilation is greater than perfusion. In high V/Q ratio areas, lower perfusion can cause some difficulties with carbon dioxide removal, while it has a minimal influence on the blood oxygenation level. However, lower perfusion in some regions of the lung releases a mechanism that directs blood to the better ventilated parts of the lungs, which leads to the development of low V/Q units in the lungs and subsequently to hypoxemia. Normalizing the V/Q ratio in low V/Q areas can be achieved when compensatory ventilation rises [21].

Complete closure of the alveoli due to atelectasis results in intrapulmonary right-to-left shunt, the extreme degree of V/Q mismatch (V/Q = 0). That means that a unit of the lungs is not ventilated and blood flows through the lungs from the right side of the heart to the left without being oxygenated. The clinical sign that distinguishes shunt from other mechanisms of hypoxemia is the lack of improvement in PaO_2_ in response to oxygen supply. In addition, intestinal gas accumulation during colic may cause increased pressure on the diaphragm, which can result in the collapse of some regions of the lungs, and therefore an increase in shunting and in the alveolar–arterial oxygen pressure difference [29]. A pulmonary shunt has been estimated to develop in 19–33% of laterally and dorsally recumbent anaesthetized horses [10]. Another type of V/Q mismatch is V/Q = ∞, which occurs when the volume of inhaled air does not take part in gas exchange, because alveoli are not perfused at all [30]. Generally, shunting and V/Q mismatch are more important than CO_2_ removal for proper O_2_ uptake from the lungs due to the shapes of their dissociation curves.

### 2.4. Dead Space

Dead space is defined as that part of a ventilated volume that does not participate in gas exchange [31]. As increased shunting impairs oxygenation, increased dead space impairs CO_2_ removal [22]. Physiological dead space (also called wasted ventilation) consists of anatomical and alveolar dead space [30]. The ratio of physiological dead space to the tidal volume is higher in horses than in other animal species [32]. In healthy lungs the physiological dead space can be considered equal to the anatomical volume. Anatomical dead space does not take part in gas exchange, but refers to the volume of air in the respiratory tract that conducts air to the bronchioles and alveoli. For clinical purposes, physiological dead space is expressed as a fraction of tidal volume (V_D_/V_T_) [31]. Regardless of how this coefficient is calculated, it assumes that all expired CO_2_ comes only from perfused alveoli [30,33]. Factors occurring during anaesthesia that influence the increase in dead space may include states of low cardiac output and the consequent decrease in pulmonary artery pressure, excessive distension of alveolar tissue during positive pressure ventilation, excessive positive end-expiratory pressure (PEEP) or pulmonary thromboembolism [30,34,35]. Mechanical ventilation is designed to counteract respiratory depression, hypercapnia and decreased blood pH during the inhalation of anaesthesia. However, the positive pressure created in the thoracic cavity may inhibit venous blood from returning to the heart, thereby decreasing cardiac output [34,35]. Those factors lead to the state where the alveoli are ventilated but not perfused. Consequently, alveolar dead space increases, which leads to an increase in the physiological dead space [33].

Physiological dead space can be estimated by using the Bohr equation and the Bohr–Engoff method. Since the modification of the Bohr equation has been used, a new component should be taken into account, especially in the case of concurrent lung diseases or severe heart failure [33]. Calculated in this way, physiological dead space is sensitive to any factor that influences the arterial–alveolar CO_2_ difference, such as venous–arterial blood mixing and other pulmonary perfusion abnormalities. This is not just a measure of dead space; it is also a general mismatch in the pulmonary V/Q ratio [30,33]. The Bohr equation is considered to be a more reliable indicator of dead space ventilation. However, it requires a mean alveolar concentration of CO_2_, but Enghoff’s modification is based on arterial CO_2_, so it is easier to obtain [30].

## 3. Influence of Hypoxaemia and Hypoxia on Haemodynamics—The Respiratory System after Recovery

Hypoxemia is defined as a state of reduced oxygen concentration in arterial blood (PaO_2_ < 60 mm Hg) that can lead to reduced oxygen levels in the tissues (hypoxia). Hypoxemia during anaesthesia can lead to organ dysfunction and post-operative complications, such as intraoperative cardiovascular disorders and post-operative multiorgan failure with clinical signs from the central nervous system, kidney, liver and myopathy [29,36]. Coronary and cerebral vasodilatation occurs during hypoxemia, while the stimulation of carotid bodies causes constriction of the visceral, pulmonary, muscular and skin blood vessels [37].

### 3.1. Haemodynamics

It has been shown that when horses inhale anaesthesia with halothane, heart rate and cardiac output increase, while vascular resistance, arterial pressure and oxygen supply to tissues decrease, either during spontaneous or mechanical ventilation [38]. Cardiovascular functions are further depressed when hypoxaemia is present during halothane-anaesthesia. It has been proven that isoflurane has a less depressive effect compared to halothane. As compensation for the hypoxia, the heart rate increases in response to decreased blood pressure (tachycardiac reflex). However, this phenomenon also increases myocardial oxygen demand, which subsequently leads to cardiac arrhythmia [39]. Moreover, during reduced haemoglobin saturation, coronary blood flow increases [38]. Studies show that the physiological signs of hypoxia (such as tachycardia) might not be apparent in some horses due to their previously described prolonged response to arterial blood gas changes [29]. In many human studies concerning hypoxia, myocardial relaxation is impaired [40]. This phenomenon is explained by the delayed removal of intracellular calcium ions, which is necessary for myocardial relaxation. When the oxygen supply is insufficient, lactate production occurs in myocardial tissues, which disturbs the metabolic, mechanical and electrical functions of the heart muscle, resulting in a decrease in contractility and thus a reduction in cardiac output [38].

### 3.2. Post-Operative Period

A persistent V/Q mismatch may increase hypoxemia in the recovery stall, which subsequently leads to life-threating complications [10,11]. Post-operative mental confusion following major surgery has been associated with hypoxemia in humans [39]. In horses, a relationship between hypoxemia and the delayed return of cognitive function has also been suggested [10,38], which may be manifested in prolonged recumbency in the recovery box. The problem may be further aggravated by muscle weakness, caused by a decrease in blood pressure [10,41]. The effect of hypoxia on the reduced strength of muscle contraction in skeletal muscles has been proven [42]. Ineffective attempts to stand during the recovery phase can increase the severity of hypoxia. There is also evidence that hypoxemia is an important risk factor for post-operative surgical site infections for horses undergoing a laparotomy [43]. Reduced oxygen partial pressure in the surgical site is a factor favouring the development of infection by weakening neutrophilic activity [44].

Intestinal hypoxia during recovery from colic surgery may occur not only as a result of ischemia but also hypoxemia [29]. In seven cases of horses undergoing a laparotomy due to colic, the authors associated the deterioration of intestinal function in the post-operative period with hypoxaemia that developed during anaesthesia. For this, they relied on studies conducted in small animals, in which hypoxemia during endotoxemia compromised intestinal oxygen extraction and uptake [45,46], and the subsequent worsening of hypoxemia increased capillary permeability [47]. The effect of low PaO_2_ levels on cardiovascular performance seemed to be greater in endotoxic horses [29].

Hypoxemia is not the only cause of hypoxia. Hypoxia can also be the result of the impaired blood flow. A recent study presented perioperative complications in a group of 1161 horses [48]. In this study, complications developed in 17.5% of horses. The most common complications were neuromuscular, accounting for 47% of all complications. Myopathies were found in 11% of affected horses and neuropathies in 19%, and their incidence was positively correlated with the weight of the horse. The mechanism of the development of post-aesthetic myositis is a decrease in muscle perfusion secondary to hypotension or an increase in pressure in the muscle fibre compartment under the pressure of the weight of the patient. An increase in pressure above capillary pressure impairs circulation, which leads to hypoxia, anaerobic metabolism, free-radical release and tissue damage [5]. The initial effect is ischemia of the muscle, and subsequently necrosis and release of toxins into the cardiovascular system.

### 3.3. Respiratory System

In the previously described study on post-operative complications, the second most common complications were respiratory in nature, with an overall incidence of 22% of all complications, 18% of which comprised pulmonary oedema. There was an association the increase in respiratory complications, a decrease in the PaO_2_/FiO_2_ ratio and an increase in the age of the animal [48]. Due to their specific anatomy, horses can only breathe through their nostrils [49]. Post-anaesthetic respiratory obstruction in the recovery period may lead to pulmonary oedema, caused by negative intrapulmonary pressure, generated during rapid attempts at breathing [48,50]. However, another important mechanism of pulmonary oedema in the perioperative period is associated with significant stimulation of the sympathetic nervous system in response to surgical stress, severe hypoxemia and hypercapnia [50]. Increased pressure and permeability in the capillaries of the lungs are the direct consequences of a hyperadrenergic condition.

Hypoxic pulmonary vasoconstriction (HPV) reduces perfusion in underventilated areas and allows blood flow to be redirected to better ventilated areas [5]. This mechanism mitigates the effects of a V/Q mismatch and shunting [28]. Systemic hypoxemia may be exacerbated when HPV is attenuated. Therefore, it is important for the anaesthesiologist to know the factors that influence this phenomenon. The protective effect of this reflex is attenuated or even abolished by volatile anaesthetic agents, although newer agents seem to have a lower influence [10,51]. It has been reported that some intravenous anaesthetics (e.g., propofol) may prevent the inhibitory effects on HPV [52]. The intensification of this phenomenon may accompany the use of high doses of lignocaine. Another factor which has a protective effect is a high-pressure ventilation with a high respiratory rate.

During general anaesthesia, the physiological mechanisms associated with the body’s response to hypoxemia are not always apparent. One reason for this is the depressive effect of inhaled anaesthetics on the respiratory response mediated by chemoreceptors [29].

## 4. Influence of Hypercapnia

Hypercapnia has two potential cardiopulmonary responses depending on the CO_2_ concentration [53]. Mild hypercapnia (PaCO_2_ = 55–65 mm Hg) during isoflurane anaesthesia with volume-controlled IPPV has a depressive effect on the cardiovascular system, causing bradycardia and contributing to a decrease in cardiac output with subsequent hypoxia, despite an increase in mean arterial blood pressure. Moreover, a significant increase in venous mixture has been noted. In the case of moderate (PaCO_2_ = 75–85 mm Hg) to severe hypercapnia (PaCO_2_ > 95 mm Hg), increased cardiopulmonary performance and the improvement of tissue oxygen supply are observed [53]. This is due to the haemodynamic changes with an increase in PaCO_2_ triggering chemoreceptor reflexes and the sympathetic adrenal response and are largely associated with an increase in plasma catecholamine concentration [53,54]. However, other studies have shown that hypercapnia increases the risk of ventricular arrhythmia [16].

Adrenergic stimulation in horses with moderately elevated CO_2_ levels may only make them appear more lightly anaesthetized, while more severe hypercapnia may cause significant depression of the CNS, which may result in reduced demand for anaesthetic agents and paradoxical respiratory depression [16].

## 5. Monitoring of Gas Disturbances

The risk factors for intraoperative complications described above can be minimized by a proper monitoring of anaesthesia and the alertness of an experienced anaesthesiologist. Intraoperative monitoring enables the assessment of changes in cardiovascular and respiratory parameters to allow early detection of disturbances during anaesthesia [4]. Many authors pointed out the need to improve anaesthetic monitoring in horses in order to reduce intraoperative mortality and post-operative complications [50,55].

Maintaining the proper level of respiratory gases depends on an appropriate gas exchange, which entails using efficient anaesthesia equipment and the proper maintenance of the cardiovascular and respiratory systems [5]. The efficiency of these two systems is assessed through the clinical examination supplemented by appropriate monitoring equipment [56]. The recommendations of the American College of Veterinary Anesthesia and Analgesia (ACVAA) for intra-operative monitoring indicate that clinical examination is sufficient for most short-term anaesthesia (<1 h) in healthy horses [57]. ACVAA guidelines recommend the use of pulse oximetry, capnography and arterial blood gas measurement for respiratory monitoring if indicated. Hypoventilation should be corrected by assisted or controlled ventilation. It is highly recommended that blood pressure be monitored during the inhalation of anaesthesia, since keeping it within the normal range (70–110 mm Hg) is essential for the proper supply of oxygen to tissues.

Monitoring the respiratory parameters is achieved by analysing oxygenation and ventilation. The normal values of the parameters listed below are presented in Table 1.

Oxygenation monitoring

- Observation of mucous membrane colour and capillary refill time
- Pulse oximetry
- Blood gas measurement
- Near infrared spectroscopy (NIRS)

Ventilation monitoring

- Observation of respiratory rate and rhythm
- Spirometry
- Blood gas measurement
- Capnography

### 5.1. Pulse Oximetry

Pulse oximetry is a method of continuous, non-invasive monitoring of the peripheral oxygen saturation of haemoglobin and pulse rate [47]. It enables the detection of hypoxaemia without having to perform complicated arterial blood gas measurements such as invasive and repeated tests since there are no continuous blood gas measurement methods [59]. It provides continuous information on the function of the cardiovascular system and on the quality of ventilation (as a late indicator of poor ventilation) but not on alveolar ventilation specifically [55]. Because the equipment requirement is minimal and taking measurements is easy, pulse oximetry is a popular monitoring method for horses during elective surgeries, emergencies and under out-of-hospital conditions [55,60].

There are two methods of pulse oximetry measurement, transmission and reflection, but both are based on the principle of spectrophotometry and the fact that oxygenated haemoglobin absorbs red light at a wavelength of 660 nm, and deoxygenated haemoglobin absorbs infrared light at 940 nm [47,55]. The amount of light reaching the detector depends on the absorption of radiation by both types of haemoglobin [47]. The degree of peripheral haemoglobin oxygen saturation is calculated by comparing the amount of oxyhaemoglobin to total haemoglobin [61].

In equine medicine, transmission pulse oximeters are commonly used. The sensor is shaped like a clip and usually placed on the tongue of horses [55]. The tongue gives the most accurate measurements [47], while the ears, nostrils, lips, vulva, and foreskin are alternative but less accurate sites. For proper signal reception between the diode and the detector, tissue thickness has to be taken under consideration because it determines the parallel placement of the sensor clips. Hair and pigmentation interfere with light transmission through the tissues [55].

In reflection oximetry, the light source and detector are placed next to each other, and the signal does not penetrate through the entire tissue, so thickness is of lesser importance [55]. With this type of transmission, it is possible to obtain reliable readings from the lips, tongue, cheek mucosa or the base of the tail in foals [55]. This type of pulse oximetry can also be used to perform measurements from the oral cavity, oesophagus or anus despite the low peripheral perfusion accompanying circulatory centralization or hypothermia [62]. However, it has not gained much popularity in veterinary medicine [63].

Studies comparing the accuracy of pulse oximetry with the measurement of blood gases have shown consistent results, although, in the case of transmission pulse oximetry, the results were overestimated in some cases, and, in the case of reflection pulse oximetry, occasionally underestimated [56].

**Figure 1:**
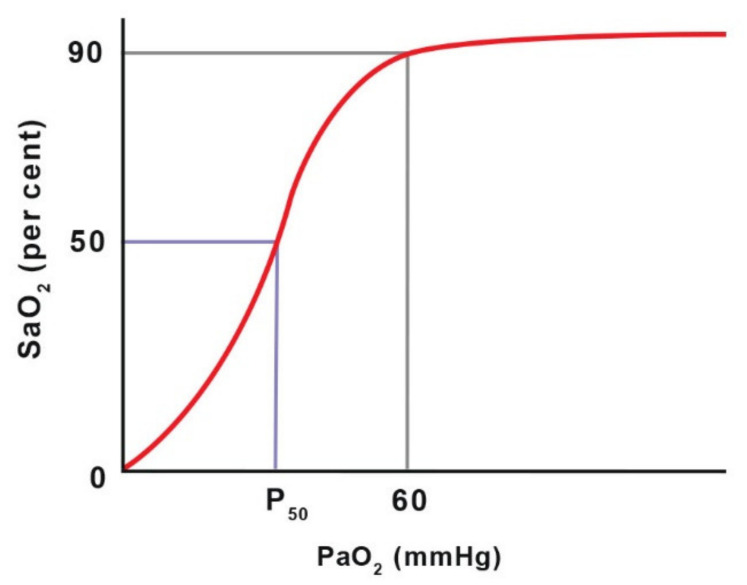
The haemoglobin dissociation curve shows the relationship between oxygen partial pressure (PaO_2_) and haemoglobin oxygen saturation (SaO_2_). P_50_ is the PaO_2_ value at which 50% of haemoglobin is saturated with O_2_ at a blood pH of 7.4 and temperature of 37 °C. Adapted with permission from [63] Copyright 2016 John Willey and Sons.

When using pulse oximeters, it is important to remember that they provide information on oxygen saturation (SO_2_), rather than partial pressure (PaO_2_). Due to the S-shape of the oxyhaemoglobin dissociation curve, significant changes in PaO_2_ can occur with only slight changes in SaO_2_ (arterial haemoglobin saturation) in the upper horizontal part of the curve [63]. Only a sudden breakdown of the dissociation curve at a relatively low PaO_2_ value (below 60 mm Hg) causes a rapid decrease in saturation. This leads to the conclusion that pulse oximetry is only useful in critical situations [63]. In this respect, blood gas measurement is better, as it sensitive to gradual changes in PaO_2_ [60]. The ability of a pulse oximeter to read low saturation values is poor, so a measurement is most reliable when SaO_2_ is above 80% [56] (Figure 1).

In patients with a low respiratory rate during oxygen supplementation, it is difficult to assess the effectiveness of ventilation, because SpO_2_ values will remain high. In such cases, a capnometer is recommended for performing blood gas analysis [64]. The device works by detecting changes in pulsating blood, so a strong regular pulse is essential for an accurate reading. In critically ill horses, measurement accuracy may be influenced by a decrease in peripheral perfusion as a result of the cardiovascular depression [62,63]. The reliability of the measurement also depends on the presence of other haemoglobin fractions, movement artifacts, vasoconstricting drug effects (including α-2-agonists), surgical light interference or electrosurgical equipment [63]. One should also reposition the sensor clip every 30–45 min, since constant pressure might impair microcirculation and give inaccurate readings [56]. The use of pulse oximeters is limited in patients with anaemia or severe acute blood loss because the device measures saturation, which would remain high despite reduced oxygen supply to the tissues [63].

Recent advances in oxygen measurement include the introduction of pulse-CO-oximeters that detect other haemoglobin fractions (dishaemoglobins). However, further studies are needed to determine whether this technique is more reliable than conventional pulse oximetry in cases of anaemia or blood loss [59]. The new generation of pulse oximeters also requires evaluation for accuracy and the elimination of the influence of movement on measurements [65,66].

In addition to saturation, pulse oximeters provide information about the pulse, and some of them display plethysmography, which is a graphic presentation of changes in the blood volume related to the cardiac cycle of superficial peripheral vessels. Cyclic changes in the amplitude of this wave during mechanical ventilation makes it possible to assess the effectiveness of fluid therapy. The use of this technique in veterinary medicine requires further research [63].

### 5.2. Near Infrared Spectroscopy (NIRS) ß

Parameters such as heart rate, blood pressure and peripheral blood oxygenation are commonly used to assess the oxygen saturation in the brain. Direct measurements of cerebral blood oxygenation are possible by measuring the partial pressure of tissue oxygen (PtO_2_) or jugular venous blood saturation (SjvO_2_), which shows the amount of oxygen in the vein after it leaves the brain. However, these methods are invasive and not routinely used in the clinical practice on horses. NIRS is a method that allows the direct and non-invasive assessment of changes in cerebral blood flow [67].

Spectroscopy is most often used to monitor cerebral blood oxygenation during general anaesthesia, but it can also be used to assess regional tissue oxygenation. In 2019, Gingold et al. published a study that showed that NIRS allows the assessment of muscle oxygenation in horses and can be useful in preventing post-anaesthetic myopathies, a serious post-anaesthetic issues [68]. There have also been reports of the use of oximetry to detect tissue hypoperfusion, which occurs in the early stage of shock, and changes in intestinal oxygenation and perfusion of the intestines during necrotizing enteritis, as was shown experimentally in piglets [69].

The infrared optical spectrum and its optical window make the skin, bones and tissues appear transparent, because the chromophores in the body (haemoglobin, myoglobin, melanin and cytochrome c) absorb the radiation [67]. Changes in radiation intensity after passing through the tissues depends on the amount of radiation the chromophores absorb [70]. The measurement is carried out using an emitter (one diode generating two light waves) and a detector (two receptor diodes). The path of the photon is elliptical and reaches a depth equal to 1/3 of the length between the radiation source and the receptor. In measuring the oxygenation of cerebral blood, photons have to pass through several layers of tissue containing different amounts of chromophores. The influence of penetrated tissues on wave absorption is minimized by two detectors. The more superficially placed receptor takes measurements from the superficial layers, and the deeper receptor takes them from deeper layers. The difference is used to calculate blood saturation [70].

**Figure 2:**
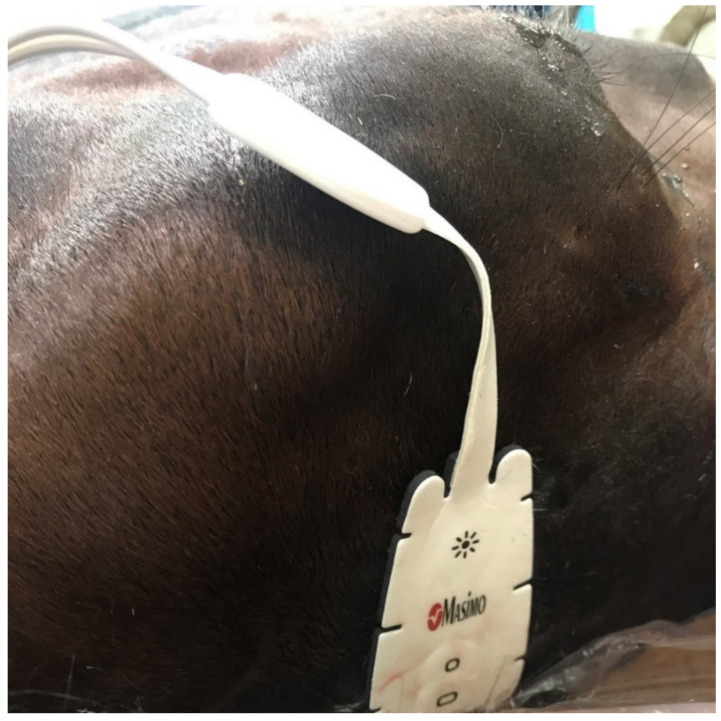
Sensor for measuring oxygenation of cerebral blood using the NIRS method (by Masimo^®^, Irvine, CA, USA), located above the dorsal sagittal sinus, which is a pool of venous blood from the cerebral hemispheres (horse is in the lateral recumbency).

In horses, self-adhesive sensors are placed on the clean, shaved skin on the midline of the forehead above the dorsal sagittal sinus, which receives the venous blood directly from the cerebral hemispheres [67]. Before the induction of anaesthesia, it is necessary to determine the baseline in relation to which the curve will be interpreted on the monitor screen and to determine the intervention threshold. The measurement is presented in real time, and the regional oxygenated to total haemoglobin ratio (rSO_2_) is presented on a scale from 0 to 100% [71,72] (Figure 2).

**Figure 3:**
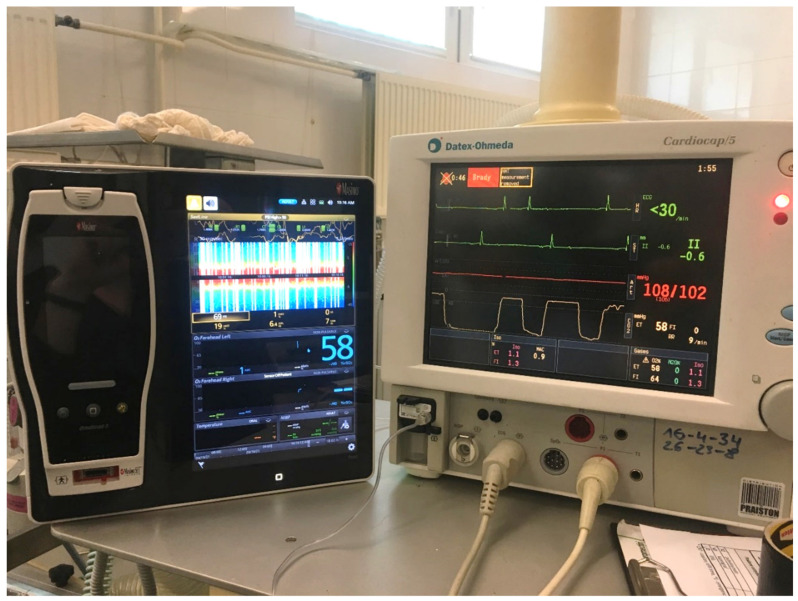
Multifunctional patient monitor (Datex Ohmeda Cardiocap S5, Helsinki, Finland), used routinely during anaesthesia (monitor on the right) and the monitor for brain oximetry (Root with Sedline, Masimo), measured with NIRS (on the left). This device also measures density spectral array.

Cerebral oximetry measures the haemoglobin oxygen saturation in the entire vascular bed, as opposed to only in the arteries as happens with pulse oximetry. It is assumed that the ratio of arterial to venous blood in the CNS is 15:85. Oximetry is therefore based on the measurement of mixed blood with a predominance of venous blood and thus enables the assessment of oxygen used by the tissues [71,73] (Figure 3).

The main problem with NIRS is the lack of a critical threshold in horses and other animals. It also has highly variable baseline values, which makes them difficult to interpret in relation to individual patients. In human medicine, rSO_2_ below 50% or a break in the reading line of more than 20% of the baseline is considered clinically significant [67]. It may indicate significant haemodynamic changes (e.g., a right-left shunt), hypotension, hypovolemia, anaemia or insufficient oxygen concentration in the hypoxic mixture. An rSO_2_ value over 85% may be associated with a high oxygen concentration in the respiratory mixture, hypercapnia or hypoglycaemia [58,71,72]. Changes in the cerebral blood flow become apparent after the autoregulation capacity of the cerebral flow is exceeded, which can be verified by measuring arterial blood pressure: in healthy adults, the limits are between 60 and 160 mm Hg [74,75].

In the study of cerebral blood oxygenation in horses [67], the same human medicine values were used as the hypothetical cut-off value, but no neurological complications or cognitive dysfunctions were found in the course of the study, which would have influenced recovery after the procedure, despite a drop below the reference value. However, as the authors emphasized, this issue requires further analysis, and the study itself confronted a number of potential limitations; rSO_2_ values being unknown for horses, which could produce perioperative complications; an arterial to venous blood ratio based on human medicine (although the measurement was performed in horses directly above the venous sinus); the thickness of the skull bones, which is different from that of humans; or the small number of tested horses.

Additionally, in this study, there was a noticeable difference in rSO_2_ values in the pre-anaesthetic and post-anaesthetic periods, which confirms the importance of gas exchange disturbances in horses during general anaesthesia. It is worth emphasizing that individual results are not as important as continuous measurement and its trends because changes indicate disorders in the blood supply to the brain. If the parameter in the animal is at a stable level from the beginning, regardless of the baseline values, it can probably be assumed that there were no disturbances in the cerebral circulation.

### 5.3. Blood Gas Measurements

Due to its high accuracy, blood gas measurement is considered the gold standard [76] and cannot be replaced by any non-invasive method [62]. It provides information about the body’s ventilation, oxygenation and metabolic status. The introduction of portable laboratory analysers, the results of which are comparable to those of reference stationary analysers, enabled their wider use in equine medicine [77]. Blood gas measurement is recommended for critically ill patients and during colic surgery [78]. Patients with gastrointestinal disorders, apart from gas exchange disturbances associated with general anaesthesia, also have disturbances in the acid–base, fluid and electrolyte balances due to the primary disorder, the evaluation of which is important for the proper assessment of therapy.

Contrary to non-invasive methods such as pulse oximetry or capnography, blood gas measurement is not continuous [60]. Its use is limited by the number of samples taken and the time needed to obtain the results; therefore, the samples are collected as needed, most often at intervals of 30 or 60 min but more frequently if the patient’s condition requires it.

**Figure 4:**
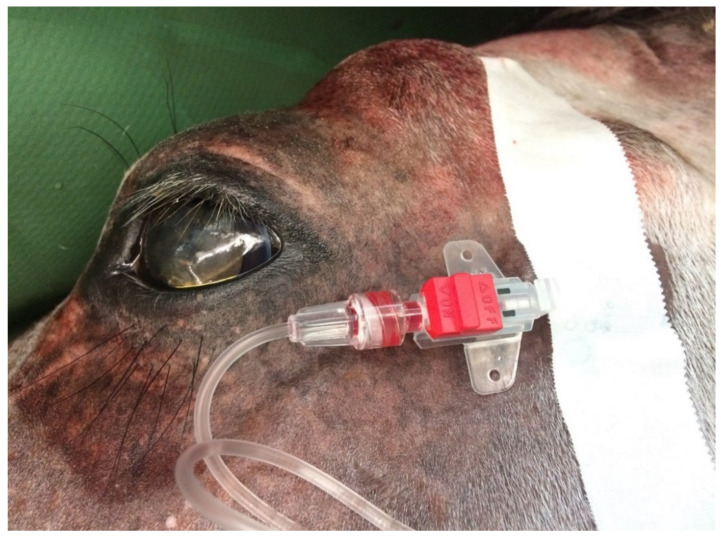
Special arterial catheter placed in the transversal facial artery, which allows one to take samples of arterial blood during a surgical procedure.

Arterial blood analysis provides information on pulmonary oxygenation capacity, ventilation and acid–base balance [27], while venous blood monitoring is only used to assess ventilation and acid–base balance. The most popular site for arterial blood collection in horses is the facial, transverse or metatarsal artery [79]. Venous blood is most often obtained from the external jugular vein [27] (Figure 4). In foals, umbilical vessels and [74], brachial, medial and dorsal metatarsal arteries are available [27,80,81]. Performing the measurement consists of taking 2–3 mL of blood (through a catheter or puncture) into heparinized syringes that are protected against air gas infiltration [60,82]. If air bubbles are present, they should be removed, and the analysis should be performed immediately after blood collection. If a delay of more than 10–15 min is expected, it is recommended to cool the sample in ice to slow the metabolism of blood cells, reduce their oxygen use and limit the production of carbon dioxide [83]. Blood samples stored on ice retain their composition stability for about 3 h after collection, while those kept at room temperature should be analysed within 10 min [40]. There are two approaches to interpreting blood gas results. The alpha-stat hypothesis assumes that it should consider normal body temperature instead of actual (core) body temperature (pH stat). As the temperature drops, pH increases and both PaCO_2_ and PaO_2_ decrease. When the temperature rises, the parameters behave in reverse. The effect of using these methods remains unclear; however, for a valid comparison, the same method should always be used [84].

The results might be biased by mistakes made before analysing the sample, such as air aspiration, excessive heparin dilution (when too small a sample was collected), delayed analysis, inappropriate storage and improper mixing, or the lack of sample temperature correction in relation to the analyser temperature [83].

### 5.4. Spirometry

**Figure 5:**
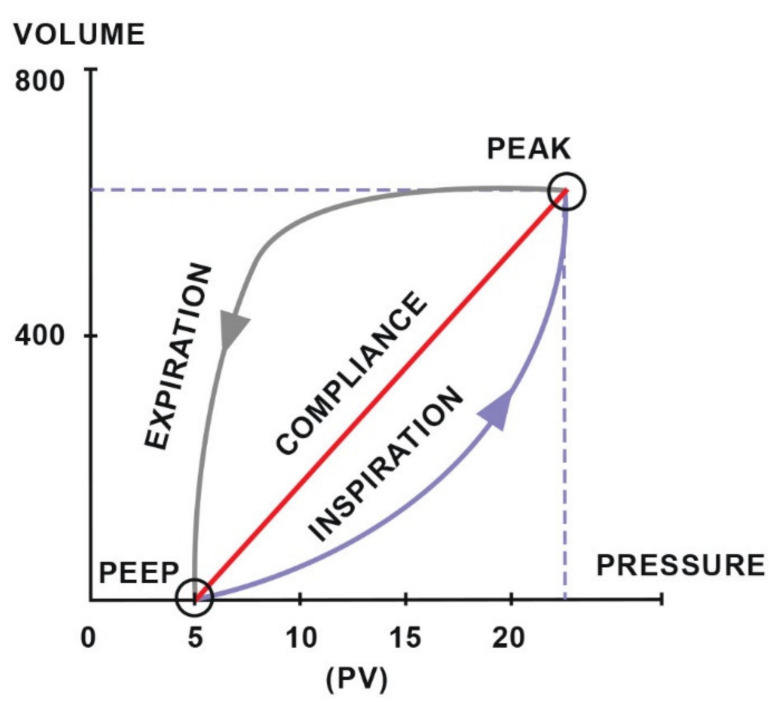
The compliance loop demonstrates the relationship between pressure and volume. Adapted with permission from [86] Copyright 2010 Y.P.S. Moens.

**Figure 6:**
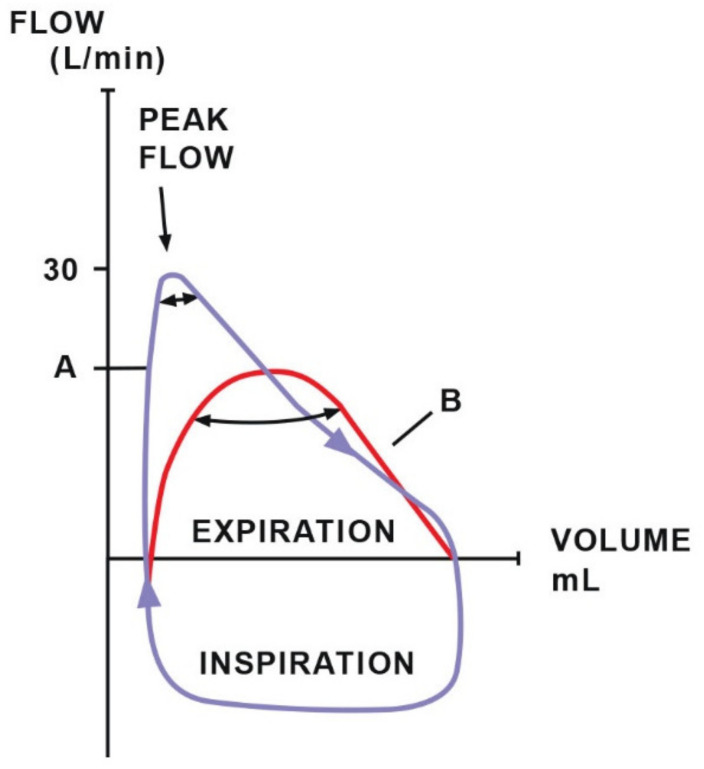
The resistance loop demonstrates the relationship between flow and volume. Loop A indicates typical resistance with a high peak flow. Loop B suggests increased airway resistance (decreased peak flow). Adapted with permission from [86] Copyright 2010 Y.P.S. Moens.

Diagnosing respiratory disorders includes the assessment of ventilation efficiency using functional tests such as spirometry [85], which measures tidal volume (TV) and minute ventilation [86]. Additionally, it provides numerical or graphical information about two important components of breathing mechanics: dynamic compliance and resistance and is a valuable supplement to pulse oximetry and capnography [85]. The data collected during one breath are visualized in the form of a pressure–volume (PV) loop, which provides information about compliance (Figure 5), and a flow–volume (FV) loop, which determines the resistance (Figure 6). The ratio of the time of inhalation and exhalation (I/E) and the volume of exhaled air during the first second of exhalation (V1.0%) describe airway resistance during exhalation [86]. Horses, compared to other animals, have an active exhalation phase at rest [20]; hence, the I/E ratio should be 1/2.

Measurements of the tidal volume, pressure and airflow in the airways are important for assessing the proper ventilation during both spontaneous breathing and mechanical ventilation [85]. The correct interpretation of the EtCO_2_ value (end-tidal CO_2_) is possible only in relation to the minute volume of ventilation [86]. In small-animal medicine, it is possible to use equipment designed for humans [87]; in horses, the incomparably high values of tidal volume, flow and generated pressure present problems [88]. However, remodelled and validated commercially available monitors with pitot tube-based flow sensors allow its wider use in large-animal practice [89].

The adaptation of the human pitot tube and the invention of monitors associated with it led to the appearance of horse spirometry on the technology market [85]. The flow sensor is placed between the tracheal tube and the breathing circuit. It also has a port for measuring respiratory gases (CO_2_, O_2_ and inhaled anaesthetics). Such a system is an important complement to the monitoring of horse respiration during anaesthesia.

It is possible to compare the loops saved in the monitor’s memory to the newly created ones. This allows easier and faster notification of irregularities and gives more time to take corrective action [85]. These measurements have many advantages. During spontaneous breathing, the respiratory rate or tidal volume indicate the depth of anaesthesia and the response to surgical stimulation. Compliance monitoring is particularly important in the case of pressure-controlled intermittent positive pressure ventilation (IPPV) [86]. In cases of low compliance, the set peak pressure may be reached before the appropriate tidal volume is delivered, resulting in hypoventilation. It remains impossible to judge this merely by observing the patient. The detection of distortions in the pressure–volume loop permits the detection of the return of spontaneous respiratory activity, which indicates insufficient depth of anaesthesia or recovery from the neuromuscular block. In the case of low compliance and pressure-controlled mechanical ventilation, the pre-set pressure can be reached before delivering the appropriate tidal volume, leading to hypoventilation and hypercapnia. When using volume-controlled ventilation, low compliance can lead to an increase in intrathoracic pressure, which generates reduced venous return, leading to reduced cardiac output. Low compliance has been seen to cause reduced lung volume because of high intra-abdominal pressure during colic, intestinal distension, gas accumulation, diaphragmatic hernia, pneumothorax or tumours [85]. Increased airway resistance seen in the flow-volume loop may indicate bronchospasm, an obstruction, a collapsed airway or a too-narrow endotracheal tube.

### 5.5. Capnography

This non-invasive method of continuous measurement of CO_2_ in exhaled air enables the assessment of alveolar ventilation, quality of gas exchange, rate of metabolism and blood perfusion through the lungs [90]. In the case of spectrophotometric analysis, the device uses the principle of absorption of infrared radiation by a sample of the flowing gas mixture [91]. The calculated absorbance difference is converted to the exhaled CO_2_ concentration (EtCO_2_). The capnometer measures the value of EtCO_2_ and displays it in numerical form as well as the capnogram (the function of changes in CO_2_ concentration over time in the form of a wave). There are also qualitative detectors of carbon dioxide available: one is based on semi-quantitative changes; the other shows a change in the colour of a filter impregnated with a pH indicator (the colorimetric method). The measurement is made in the sidestream or main breathing stream, and the first method has gained the greatest popularity for horses [60]. In the mainstream measurement, the sensor is placed between an endotracheal tube and a breathing circuit. This location shows the partial pressure of CO_2_ in real time and does not disturb the capnogram because there is no dispersion of gases. However, mainstream capnometers are more accurate in patients with a small tidal volume and are not useful for larger animals because of the large-diameter endotracheal tubes and because coupling connectors are required for mainstream measurement [92]. The sidestream system requires transporting the respired gases through a sampling tube to the sensor located in the sampling cell within the monitor. For this reason, there is a reading delay and the possibility of leakage and obstruction at any of the numerous connection points in this system [93].

**Figure 7:**
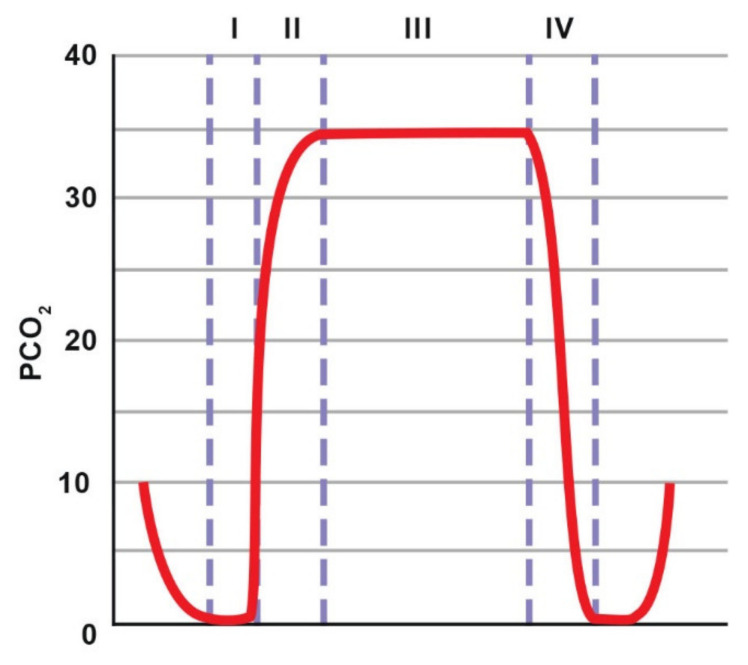
Normal capnogram. The description is included in the text. Adapted with permission from [91] Copyright 2013 Elsevier.

The assessment of the capnogram made it possible to identify problems with the patient or equipment that might otherwise go unnoticed [91]. The graph of the breathing cycle starts at the beginning of inspiration and ends at the end of exhalation (Figure 7). Phase 1 corresponds to the beginning of expiration, at which stage air is exhaled from the dead space of the respiratory tract and breathing circuit; therefore, the mixture of exhaled gases contains at most only a small amount of CO_2_. Phase 2 reflects the transition of air from the dead space to the alveoli for gas exchange; therefore, a sharp increase in CO_2_ concentration is observed. Phase 3 is the plateau phase with an almost constant gas concentration corresponding to alveolar air. In phase 4, CO_2_ concentration drops, indicating the beginning of the next inspiration. A gradual increase in EtCO_2_ may indicate hypoventilation or an increase in metabolism (sepsis, malignant hyperthermia). A drop in EtCO_2_ may also occur in the event of disconnection from the circuit, gas leakage, pulmonary embolism or cardiac arrest.

The ACVAA guidelines for intraoperative monitoring of the respiratory system in horses emphasize that EtCO_2_ often underestimates PaCO_2_ values [94]. This underestimation in large animals is much greater than for small animals, which is explained by particularly strong disturbances in gas exchange such as atelectasis and perfusion-ventilation mismatch. Additionally, the diameter of the upper respiratory tract in a horse is larger, which results in diluted alveolar air in the air from the dead space [60]. Therefore, EtCO_2_ measurement should not replace the direct measurement but should be treated as an additional one that allows for a continuous overview of changes. It does, however, reduce the number of samples that need to be taken [57,95]. For procedures lasting less than 60 min, at least one blood gas analysis should be performed to assess the consistency of EtCO_2_ and PaCO_2_ measurements and should be repeated 60 min after the procedure. It should be remembered that disturbances of gas exchange in horses deepen with the duration of anaesthesia; therefore, a prolonged procedure will increase the discrepancy between the values.

Increased EtCO_2_ may be the result of hypoventilation, malignant hyperthermia, fever or sepsis. A gradual decrease in EtCO_2_ may be caused by hyperventilation, hypothermia or declining cardiac output. A sudden drop in EtCO_2_ may be a result of extubation, equipment failure, massive pulmonary embolism, or cardiopulmonary arrest [91].

EtCO_2_ measurement is helpful for deciding whether to use a mechanical ventilator to regulate breathing frequency [95]. Capnography also allows for the quick capture of the moment of apnoea that occurs in horses under the influence of anaesthetic drugs. Capnometry is a useful tool in cardiopulmonary resuscitation because CO_2_ delivery to the lungs requires adequate pulmonary circulation [91]. An increase in exhaled CO_2_ indicates that the chest compressions are deep enough to ensure adequate stroke volume or that spontaneous breathing has recovered. Additionally, regardless of changes in numerical values, the interpretation of the shape of the capnogram allows for the diagnosis of many clinically significant problems. An elevation of the baseline (if a baseline does not reach 0) indicates the rebreathing of CO_2_, which may be caused by damaged valves in the breathing circuit or used soda lime. Loss of the alveolar plateau (phase III in a normal capnogram) may be associated with a pneumothorax. Moreover, when mechanical ventilation is used, a conflict between imposed breaths (directed by the ventilator) and spontaneous breaths (patient-ventilator asynchrony or buckling) can be identified [91,96,97]. A notch (curare cleft) may be seen in cases when a patient receives a neuromuscular blocking agent and is being ventilated mechanically. The notch indicates that the patient is making an attempt to breathe when the effect of neuromuscular blocking agents is wearing off. Another very characteristic waveform change is the so-called “shark fin”, which occurs in the case of airway obstruction [91,97].

## 6. Summary

Significant changes in pulmonary function, haemodynamics and gas exchange seem to be the cause of numerous post-operative complications and presumably contribute to high post-operative mortality in horses. The use of specialized monitoring equipment provides valuable information about the condition of the anaesthetized patient. Improving the methods of anaesthesiologic supervision, proper interpretation of the results, understanding the limitations of the equipment and understanding the factors that may affect the reading of monitoring devices will contribute to the safety of anaesthetized horses.

## Figures and Tables

**Table 1:** Normal physiological values for anaesthetised horses (according to [44,51,58]).

**Function of the System**			**Measurement Method**	**Parameters Measured**	**Normal Values**
	Respiratory	Ventilation	Clinical exam	Respiratory rate (breaths/minute)	6–20
			Spirometry	Tidal volume (mL/kg)	10
				Resistance (cm H_2_O/L/s)	<1.2
				Compliance (L/cm H_2_O)	2.4
			Blood gas	Arterial blood pH PaCO_2_ (mm Hg)	7.30–7.45 40–60
			Capnography	EtCO_2_ (mm Hg)	30–50
	Respiratory and cardiovascular	Oxygenation	Pulse oximetry	SpO_2_ (%)	93–100
			Blood gas	PaO_2_ (mm Hg)	100–500 (depending on FiO_2_)
			NIRS	O_3_ (%)	55–85

## Data Availability

Not applicable.
